# Supplementary material for: The Mutation of the DNA-Binding Domain of Fur Protein Enhances the Pathogenicity of Edwardsiella piscicida via Inducing Overpowering Pyroptosis
Source: Microorganisms. 2023 Dec 20;12(1):11. doi: 10.3390/microorganisms12010011 (PMC10821184; doi:10.3390/microorganisms12010011)
Supplement: Supplementary file 1 [file microorganisms-12-00011-s001.zip › microorganisms-2700488-supplementary.pdf]

The Mutation of the DNA-Binding Domain of Fur Protein Enhances the Pathogenicity of *Edwardsiella piscicida* via Inducing Overpowering Pyroptosis

Mimi Niu, Zhihai Sui, Guoquan Jiang, Ling Wang, Xuemei Yao, Yonghua Hu

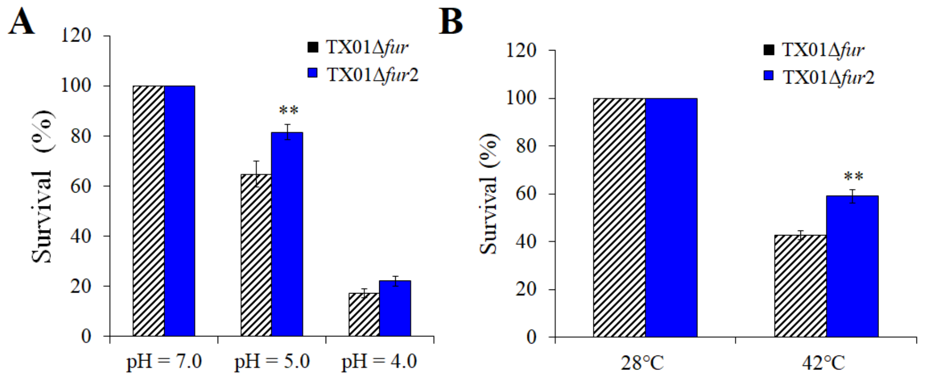

Figure S1. Sensitivity of *Edwardsiella piscicida* to environmental stresses. (A) TX01Δ*fur* and TX01Δ*fur*2 were incubated in LB medium of pH 4.0, 5.0 and 7.0 for 4 h, respectively. The populations of survival bacteria were counted by diluted plate and the survival rate was calculated. (B) TX01Δ*fur* and TX01Δ*fur*2 were incubated in LB medium for 4 h at 28 °C and 42 °C, respectively. After washing, the populations of survival bacteria were counted by diluted plate and the survival rate was calculated. Data are the means of three independent experiments and presented as mean ± SEM.*, $P < 0.05$; **, $P < 0.01$.

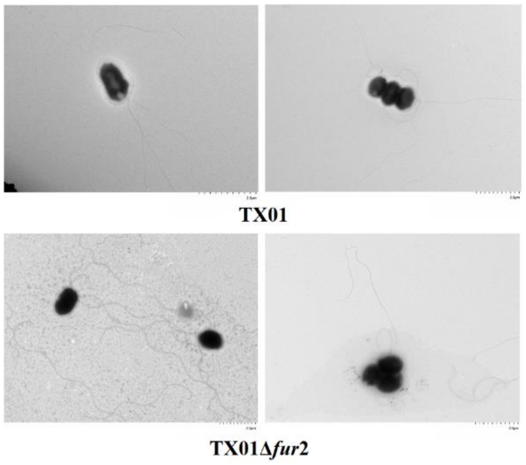

Figure S2 The flagella observation by TEM. TX01 and TX01Δ*fur*2 were cultured in LB solid medium. Bacteria were resuspended with PBS and stained by 1% phosphotungstic acid, flagella were observed using the TEM.
